# Hyaluronan Hydrogels: Three Material States in Regenerative Orthopaedics

**DOI:** 10.3390/gels12100867

**Published:** 2026-09-25

**Authors:** Fábio Ramos Costa, Maria Helena Andrade Santana, Rubens Martins de Andrade, João Protásio Netto, Luyddy Pires, Gabriel Azzini, André Kruel, Tomas Mosaner, Lucas Furtado da Fonseca, José Fábio Lana

**Affiliations:** 1 Department of Orthopedics, FC Sports Traumatology, Salvador 40296-210, BA, Brazil; 2 Department of Orthopedics, Brazilian Institute of Regenerative Medicine (BIRM), Indaiatuba 13334-170, SP, Brazil; luyddypires@gmail.com (L.P.); josefabiolana@gmail.com (J.F.L.); 3 Regenerative Medicine, Orthoregen International Course, Indaiatuba 13334-170, SP, Brazil; drgabriel.azzini@gmail.com (G.A.); kruel.andre@gmail.com (A.K.); tmosaner@uol.com.br (T.M.); ffonsecalu@gmail.com (L.F.d.F.); 4 Department of Engineering of Materials and Bioprocesses, School of Chemical Engineering, University of Campinas, P.O. Box 6066, Campinas 13083-852, SP, Brazil; mariahelena.santana@gmail.com; 5 Medical School, Tiradentes University Center, Maceió 57038-000, AL, Brazil; rubensdeandrade@hotmail.com; 6 Department of Orthopedics and Traumatology, Federal University of Tocantins (UFT), Palmas 77001-090, TO, Brazil; jprotasionetto@gmail.com; 7 Medical School, Max Planck University Center (UniMAX), Indaiatuba 13343-060, SP, Brazil; 8 Clinical Research, Anna Vitória Lana Institute (IAVL), Indaiatuba 13334-170, SP, Brazil; 9 Medical School, Jaguariúna University Center (UniFAJ), Jaguariúna 13911-094, SP, Brazil

**Keywords:** hyaluronic acid, hydrogel, cross-linking, viscosupplementation, rheology, osteoarthritis, regenerative medicine, BDDE, HYADD-4

## Abstract

Hyaluronan-based materials are widely described as hydrogels, yet the term is applied without distinction to preparations that differ fundamentally in structure. This review addresses that imprecision by separating three material states of hyaluronan and by stating the criteria under which each may be called a gel: native linear hyaluronan, which forms a viscoelastic solution; the hexadecylamide derivative (HYADD-4), which self-assembles into a transient physical network through hydrophobic interactions; and hyaluronan cross-linked with 1,4-butanediol diglycidyl ether (BDDE), which forms a covalent three-dimensional network. A material is treated as a gel here only where both a percolated network and a solid-like mechanical response are documented. On that basis the covalent network qualifies; the derivative is elastic-dominated across the walking-to-running frequency band of the knee but has not been subjected to a formal Winter–Chambon analysis and is therefore described as gel-like; and the linear solution does not qualify at the molecular weights and concentrations used in marketed viscosupplements. We review the chemistry of network formation, the comparative physicochemical and rheological behavior, including viscosity, the storage and loss moduli, boundary lubrication, and resistance to enzymatic and oxidative degradation, and the receptor-mediated and anti-inflammatory mechanisms of action involving CD44 and molecular-weight-dependent signaling. Clinical and regenerative applications in osteoarthritis viscosupplementation and in scaffolds and carriers for cells and growth factors are summarized for each state. The networked materials show higher viscoelasticity and greater resistance to degradation than the linear solution, which provides a rationale for longer residence; the evidence for a clinical advantage over conventional hyaluronan, however, rests largely on preclinical and rheological data. A three-state framework is proposed, pending validation, to support clearer interpretation of the literature and more rational material selection.

## 1. Introduction

Hyaluronan is a linear, non-sulfated glycosaminoglycan composed of repeating disaccharide units of D-glucuronic acid and N-acetyl-D-glucosamine, and it is distributed throughout the connective tissues and body fluids of vertebrates, where it contributes to tissue hydration, matrix organization, and the lubrication of articulating surfaces [1]. In its native state, the polymer exists as a high-molecular-weight chain that adopts an expanded random-coil conformation in solution; the resulting entanglement and water retention give synovial fluid its characteristic viscoelasticity, yet the native polymer remains a viscous solution rather than a structured solid [1]. This distinction between a viscous solution and a structured network is central to the present review.

Over the past three decades, hyaluronan has been adapted into a range of physical forms for clinical use, from intra-articular viscosupplements for osteoarthritis to scaffolds and carriers in regenerative procedures [2,3]. Chemical modification of the polymer can transform it from a viscoelastic solution into soft or rigid hydrogels, electrospun fibers, sponges, and particulate fluids, each with distinct mechanical and biological behavior [3]. The term hydrogel, defined as a hydrophilic polymeric network capable of absorbing and retaining substantial volumes of water without dissolving, is frequently applied across these forms without regard to the nature of the underlying network [2]. The consequence is a terminological imprecision in which a native hyaluronan solution, a physically associated network, and a covalently cross-linked matrix are all described interchangeably as hydrogels, despite marked differences in structure and function.

Intra-articular hyaluronan preparations are widely used for symptomatic knee osteoarthritis, and a body of preclinical and clinical work has examined how molecular weight, concentration, and network architecture influence their residence time and biological activity [4]. These variables are not interchangeable, and the mechanism through which a given preparation acts depends on whether it behaves as a lubricating solution, a transient physical network, or a durable covalent network. Clarifying this dependence is relevant both to the interpretation of comparative clinical data and to the rational design of next-generation materials.

This review is organized around the proposition that hyaluronan-based materials should not be treated as a single category. We distinguish three material states: native linear hyaluronan, which forms a viscous solution; the hexadecylamide derivative of hyaluronan (HYADD-4), which self-assembles into a physical, non-covalent network through hydrophobic interactions; and hyaluronan cross-linked with 1,4-butanediol diglycidyl ether (BDDE), which forms a covalent three-dimensional network through ether linkages. We examine the chemistry of network formation, the comparative physicochemical and rheological behavior, the receptor-mediated and anti-inflammatory mechanisms of action, and the clinical and regenerative applications of each state, with the aim of providing a coherent framework for interpreting the literature and for guiding material selection in orthopaedic and regenerative practice.

### Criteria Applied in This Review for Treating a Hyaluronan Preparation as a Gel

Because the term gel is applied inconsistently across the hyaluronan literature, the criteria used in this review are stated at the outset rather than left implicit. A preparation is treated here as a gel only where two conditions are documented for that preparation. The first is structural: a network of junctions that percolates the sample, in the sense of Flory and Stockmayer, rather than a dispersion of finite clusters or an entangled but unconnected solution [5,6]. The second is mechanical: a solid-like response demonstrated rheologically, with the formal signature given by Winter and Chambon, namely parallel power-law scaling of G′ and G″ and a loss tangent independent of frequency [7,8]. A third consideration, the Deborah number, fixes the observation window over which any such response is assessed but does not by itself demonstrate either condition [9]. Applied to the three material states, these criteria are satisfied in full by the covalently cross-linked network; they are not satisfied by native linear hyaluronan at the molecular weights and concentrations used in marketed viscosupplements; and they are satisfied only in part by the hexadecylamide derivative, which is elastic-dominated across the working frequency band of the knee but for which no formal Winter–Chambon analysis has been published. The derivative is therefore described throughout as a transient physical network rather than as an established gel. Section 5 develops each criterion in turn, and Section 5.5 sets out which experimental methods can and cannot be used to establish them.

## 2. Review Methods

This work is a structured narrative review rather than a systematic review, and it does not follow PRISMA reporting or include a quantitative meta-analysis. Literature was identified through searches of PubMed, EMBASE, and Web of Science combining the terms hydrogel, hyaluronic acid, cross-linked hyaluronic acid, BDDE, HYADD-4, hexadecylamide hyaluronan, viscosupplementation, rheology, intra-articular, boundary lubrication, and CD44. The final search was run on 9 June 2026. No lower limit was placed on publication date; priority was given to records published in the ten years preceding that date, and earlier work was retained where it establishes the physical criteria applied here. Records were eligible if written in English and if they reported chemistry, physicochemical or rheological characterization, degradation behavior, receptor biology, or intra-articular or regenerative use of hyaluronan in one of the three material states. Records were excluded where no full text was accessible, where the item was a conference abstract or had not been peer-reviewed, or where the indication fell outside the musculoskeletal and regenerative scope of this review; dermal-filler studies are a deliberate exception, retained only where they provide physicochemical characterization of BDDE networks. Records retrieved from the three databases were merged and deduplicated by digital object identifier, and by title with first author and year where no identifier was registered. Studies were assigned to a material state according to the chemical identity of the material tested as reported by the source, and not by inference from the rheological behavior reported. Because the literature specific to the hexadecylamide derivative is limited in volume, each retrieved record relating to that material was assessed individually for relevance and accuracy. One regulatory and manufacturer source is cited: the package insert of the marketed HYADD-4 product [10]; it supplies formulation, labeling, and dosing information only, is identified as a regulatory source at each point of use, and is not treated as peer-reviewed evidence of efficacy. Reference details, including digital object identifiers where available, were verified against the source databases.

## 3. What Is a Hydrogel? Defining the Three Material States

A hydrogel is a network of hydrophilic polymer chains that imbibes and holds a large volume of water while resisting dissolution, a property that derives from the presence of junctions between chains that limit their dispersion [2]. The composition of the network, the character of the bound and free water, and the permeation of solutes within the swollen matrix together determine the suitability of a given hydrogel for drug delivery, cell encapsulation, and tissue engineering [2]. The defining feature is the junction: without inter-chain connections that persist on the relevant timescale, a hydrophilic polymer in water remains a solution.

Hydrogels are commonly classified by the nature of these junctions [11]. Physical hydrogels are stabilized by reversible interactions such as hydrophobic association, hydrogen bonding, electrostatic attraction, and chain entanglement, and their networks can dissociate in response to changes in the local environment [11]. Chemical hydrogels are stabilized by irreversible covalent bonds introduced through cross-linking reactions, which produce networks of higher junction density and greater resistance to dissolution [11]. This division provides the conceptual axis for separating the three hyaluronan states considered here.

In the first state, native linear hyaluronan is dissolved in an aqueous medium and forms a viscoelastic solution whose properties arise from chain entanglement and water retention rather than from stable junctions [1]. In the second state, the hexadecylamide derivative of hyaluronan carries a small number of C16 alkyl side chains that associate through hydrophobic interactions, producing an injectable physical network without covalent cross-linking [12]. In the third state, hyaluronan reacted with BDDE under alkaline conditions acquires covalent ether linkages between chains, yielding a true three-dimensional covalent network [13]. The structural and bonding distinctions among the three states are summarized in Figure 1 and Table 1.

The three material states are presented as representative archetypes of three network conditions, no persistent network, a reversible physical network, and an irreversible covalent network, rather than as an exhaustive catalogue. Within the covalent, non-reversible category, BDDE cross-linking is taken as the representative chemistry, although clinically established alternatives exist, including divinyl sulfone–cross-linked hylan G-F 20, NASHA, photo-cross-linked hyaluronan (Gel-One), and the benzyl-ester scaffold HYAFF-11. They are noted here to situate the three states within the broader landscape of hyaluronan-based networks, without implying that either modified category is confined to a single chemistry. The chemistries underlying these products, namely divinyl sulfone, epoxide (BDDE), and benzyl esterification, are reviewed in [14].

## 4. Chemistry and Network Formation

### 4.1. Native Linear Hyaluronan

Native hyaluronan is produced by bacterial fermentation or extracted from animal tissue and is supplied as an unmodified linear polymer [4]. In aqueous solution the chains do not form stable junctions, and the material behaves as an entangled solution whose viscosity rises with molecular weight and concentration [1]. The absence of covalent or persistent physical junctions means that the polymer is susceptible to rapid clearance and enzymatic and oxidative degradation once introduced into a joint, a point developed in Section 6 [1].

### 4.2. Hexadecylamide Derivatization (HYADD-4)

The hexadecylamide derivative of hyaluronan is prepared by amidation of a fraction of the carboxyl groups along the polymer backbone with hexadecylamine, introducing a low number of C16 alkyl side chains per chain [12]. The parent polymer used in the published characterization is a bacterial sodium hyaluronate of 7 × 10^5^ Da, converted to the tetrabutylammonium salt, activated with 1,1′-carbonyldiimidazole, and amidated with hexadecylamine at 42 degrees C [12,15]. Gel permeation chromatography of substrate and product indicates that the molecular weight of the polysaccharide is almost unchanged by the grafting reaction, so that the altered viscoelastic behavior is attributable to the side chains rather than to chain length [12]. The degree of substitution is low, between 1 and 3 mol per 100 repeating units, and the batches used for physicochemical and rheological characterization carried 2.4 mol% [12,15]. The marketed formulation is an 8 mg/mL (0.8% *w*/*v*) dispersion of the derivative in phosphate-buffered saline at pH 6.9, autoclaved to yield a clear hydrogel [10,12]. A self-supporting network is obtained at 3 mg/mL (0.3% *w*/*v*) and above, whereas the unmodified parent polymer forms viscous solutions only at approximately ten times that concentration [12,15]. Dynamic light scattering of these dispersions gives an average network correlation length falling from 24 nm at 3 mg/mL to 13 nm at 8 mg/mL, with characteristic relaxation times falling from about 210 to 85 microseconds across the same range [12,15]. The oscillatory measurements discussed in Section 5.2 were performed on a stress-controlled rheometer with a 20 mm parallel-plate geometry and a 500 micrometre gap at 25 degrees C, within a linear viscoelastic region extending from about 0.1% to 50% strain, and the authors report no meaningful difference between room temperature and 37 degrees C; the frequency sweeps were referred throughout to the walking-to-running band of the knee, 0.5 to 3 Hz [12]. An explicit determination of the overlap concentration c* for this derivative has not been reported in the primary literature, so the semidilute assignment made below rests on the network parameters above rather than on a measured c*, and that gap is stated here rather than filled by inference. These hydrophobic side chains associate in aqueous medium, and the resulting inter-chain hydrophobic interactions generate a physically associated network that is stabilized without any covalent cross-linking [12]. Because the junctions are non-covalent, the network can recover after deformation and remains injectable, while still resisting dispersion more effectively than the unmodified solution [12]. The ability of these associations to constitute a network depends critically on the concentration regime of the derivative. In the dilute regime, where the chains are isolated, the C16 side chains associate preferentially in an intramolecular fashion, forming loops within a single chain that compact the coil without establishing a percolating network or cooperatively raising the elastic modulus. It is in the semidilute regime, above the overlap concentration c* [16,17], where the chains are already interpenetrated, that the associations become predominantly intermolecular, or interchain bridges, and the physical network is established. The semidilute regime is therefore not merely favorable to network formation but the condition that renders it possible. The low degree of hydrophobic substitution of the derivative is consistent with this mechanism, favoring bonds between different chains, which keep the network loose and injectable, rather than the collapse of each chain onto itself that a high number of side chains would tend to produce.

Once established, however, this network is finite in permanence, precisely because its junctions are non-covalent and exist in dynamic equilibrium with the unassociated chains. It can be disrupted by sufficient shear, progressively dissociated upon dilution as the equilibrium shifts toward the free state, and modulated by temperature, since the strength and lifetime of hydrophobic associations are temperature-dependent. These sensitivities distinguish the physical network from a covalent one, which is not disrupted by dilution or shear. They are also important within the articulation, where the material is exposed to both dilution by synovial fluid and mechanical load.

### 4.3. BDDE Cross-Linking

Cross-linking of hyaluronan with BDDE proceeds under alkaline conditions, where the epoxide groups of the bifunctional reagent undergo ring-opening reactions with hydroxyl groups on the polymer to form ether linkages, producing a covalent three-dimensional network [13]. The degree of modification, the molecular weight of the starting polymer, and the reaction conditions all influence the swelling, rheology, cohesivity, and enzymatic resistance of the resulting gel [13]. A relevant feature of the process is that the strongly alkaline environment also promotes hydrolytic depolymerization of the polymer during cross-linking, so that differences in starting chain length are partially attenuated in the final product [13]. Lowering the reaction temperature increases the ratio of cross-linking to hydrolysis, which raises the storage modulus, reduces pore size, and enhances enzymatic resistance, illustrating how the balance of competing reactions governs the properties of the network [18].

The concentration regime in which cross-linking is carried out also modulates the final elastic modulus. When the polymer is cross-linked in the semidilute regime, the interchain entanglements that covalent cross-linking traps contribute to the equilibrium modulus in parallel with the covalent junctions, so that a given modulus target can be reached with a lower cross-linker content. It is important, however, to distinguish this gain in modulus from the location of the gel point: being transient, entanglements do not percolate a permanent network and do not shift the gel point, which is fixed by the conversion of covalent bonds; they raise the post-gel modulus rather than advancing gelation. This contribution is also bounded, since the hydrolytic depolymerization and reaction conditions described above raise the overlap concentration and erode the entanglements that would sustain it, which is consistent with this window being limited rather than open-ended.

The covalent network formed with BDDE is generally characterized by an elastic, solid-like rheological profile and a fibrous internal architecture, which distinguishes it from both the unmodified solution and the physically associated derivative [19]. The choice of cross-linker concentration entails a balance, since residual cross-linker and reaction by-products have been raised as potential safety considerations in the dermal-filler literature, and approaches using minimal cross-linker content have been explored to address this concern [20,21]. These chemical considerations, summarized for the derivatized and cross-linked states in Figure 2, set the basis for the physicochemical differences described next.

## 5. Physicochemical and Rheological Properties

### 5.1. Molecular Weight, Concentration, and Viscosity

The viscosity of a native hyaluronan solution depends on the molecular weight and concentration of the polymer, with longer chains and higher concentrations producing greater entanglement and higher apparent viscosity [1]. Because the unmodified polymer lacks stable junctions, its solutions exhibit a concentration-dependent viscoelasticity but do not display the frequency-dependent gel behavior characteristic of a network [12]. Both the hexadecylamide derivative and the BDDE network achieve higher apparent viscosity and shear-thinning behavior at comparable polymer concentrations through their respective junctions, rather than through chain length alone [12,13].

### 5.2. The Rheological Gel Criterion: Storage and Loss Moduli Across the Three States

The question posed in the title of this review has a precise physical answer: under the criteria that characterize the gel state, native linear hyaluronan is not a gel at the molecular weights and concentrations used in marketed viscosupplements, the BDDE network is a gel across the entire range of frequency and timescale examined, and HYADD-4 behaves as a transient physical network whose solid-like character is documented across the working frequencies of the knee but whose formal gel status has not been established. Reaching that answer requires making precise a term that the literature applies loosely: “gel” is not a synonym for viscous or thick but denotes a state of matter separated from the liquid by a defined transition. Three physical criteria render this distinction operational. They are complementary and not interchangeable, and each settles a different question; the rheological criterion provides the most direct mechanical signature.

The rheological criterion (Winter–Chambon) is the mechanical signature of the gel. The storage modulus G′ measures the elastic response of the material and the loss modulus G″ the viscous one. At the gel point, the two quantities come to scale with the same power of frequency (G′, G″ ∝ ω^n^, with 0 < n < 1), so that their ratio, the loss tangent, tan δ = tan(nπ/2), becomes independent of frequency [7]. It is this frequency independence, rather than the simple crossover of the two curves, that is the fingerprint of the gel: the crossover of G′ and G″ coincides with the gel point only when n = ½, and for any other value it occurs before or after gelation [8]. Because n is a property of the material and has not been determined for HYADD-4, the crossover frequency reported for that material cannot be read as a gel point; the robust criterion is parallelism with a constant tan δ. The crossover is used in this review only as a practical landmark separating viscous-dominated from elastic-dominated behavior.

To this mechanical criterion two adjacent ones are added. The percolation criterion (Flory–Stockmayer) provides the structural definition: above a critical density of junctions a single network spanning the entire sample is formed, whereas below it the material remains a sol of disconnected fragments [5,6]. The timescale criterion (Deborah number) provides the temporal relativity: De = λ/t_obs compares the relaxation time of the material with the time over which it is observed, with a solid-like response for De > 1 and a liquid-like one for De < 1 [9]. These three statements are logically distinct and answer different questions. The Winter–Chambon criterion identifies the mechanical signature of the transition, the Flory–Stockmayer criterion states the structural condition for a percolated network, and the Deborah number relates a relaxation time to an observation time. A high Deborah number is therefore not independent evidence of gelation: an entangled solution with a long relaxation time responds as a solid over a short observation window without possessing a percolated network of persistent junctions. In what follows the Deborah number is used only to describe how stable the solid-like response of HYADD-4 is within the frequency window of the joint, and never in place of the structural or the mechanical criterion. The nature of the junctions, reversible in HYADD-4 and covalent in the BDDE network, completes the distinction between a physical and a chemical gel.

From these criteria a central point follows: the mechanical state observed is not a constant of the material but depends on two conjugated factors, on the biomaterial as produced and on the regime in which it is applied. Production fixes the intrinsic properties (the relaxation time λ and the exponent n), governed, in the BDDE network, by the cross-link density, the degree of modification, and the reaction conditions, and, in HYADD-4, by the degree of hydrophobic substitution. Among these production conditions the concentration regime is decisive: when the material is obtained in the semidilute regime, chain overlap contributes of itself to the moduli, through trapped entanglements in the BDDE network and through the conversion of intramolecular loops into interchain bridges in HYADD-4, so that a given G′ target can be reached with less cross-linker or a lower degree of hydrophobic substitution (Section 4). Application fixes the observation timescale, which has two dimensions: the frequency band that the cartilage experiences, which is not single but extends from walking to running [22], and the intra-articular residence time, the long timescale over which the material persists and over which dilution and degradation alter λ itself. The mechanical state of a viscosupplement is therefore evaluated at the meeting of a material of given λ with a band of frequencies and times imposed by use.

Under this framework the three states are distinguished, each with the degree of confidence its data support. Native linear hyaluronan, dominated by the viscous response and without a percolating network of persistent junctions, does not meet the gel criteria at the molecular weights and concentrations used in marketed linear viscosupplements, which are the formulations covered by the studies cited here [1,4,12]. That restriction is deliberate. The response of uncross-linked hyaluronan depends on molecular weight, concentration, ionic environment, and degree of chain overlap, and sufficiently concentrated, highly entangled solutions can display a predominant elastic response over particular frequency windows through entanglement alone; such behavior reflects a long relaxation time rather than percolation and does not satisfy the formal criterion. The BDDE network satisfies the structural and mechanical criteria across the whole range examined and resists degradation, maintaining a high λ throughout residence. HYADD-4 occupies an intermediate position that rests on weaker evidence. In direct comparison with marketed viscosupplements, the derivative formulated at 8 mg/mL shows G′ above G″ throughout the 0.5–3 Hz band of the knee, its crossover frequency lying below that band rather than within it, and it is the only non-covalently cross-linked hyaluronan product reported to show this elastic-dominated profile in that window [12]. Because a physical network becomes increasingly elastic-dominated as the loading frequency rises, this character is sustained across the higher frequencies of running and heel-strike [22], where the Deborah number rises further above unity. Over long observation times at rest the material flows as a liquid, since the Deborah number falls below unity. Injectability, by contrast, arises outside the linear regime to which these criteria apply: the high shear rates developed within the needle disrupt the reversible hydrophobic junctions and lower λ itself, so that the network yields during delivery and reforms after deposition, a recovery that has been measured directly and is complete within a few hundred seconds after repeated mechanical shock [12]. The solid-like character of HYADD-4 is therefore conditioned twice over, by the frequency band imposed by the joint and by the amplitude of deformation imposed by use, rather than being a fixed property of the material. Decisively, a formal Winter–Chambon analysis of the derivative, in which parallelism of G′ and G″ with a frequency-independent tan δ is demonstrated directly, has not been reported. The derivative is therefore described throughout this review as a transient physical network, or gel-like, rather than as an established gel, and such an analysis remains the most direct target for future characterization. These relationships are represented schematically in Figure 3.

### 5.3. Tribology and Boundary Lubrication

The tribological behavior of intra-articular hyaluronan is distinct from its bulk rheology and merits separate consideration. Healthy synovial joints sustain very low friction coefficients, reported to approach values on the order of 0.001, through a combination of fluid-film and boundary lubrication, with the boundary contribution attributed to hydration lubrication and to interactions among hyaluronan, lubricin, and surface-active phospholipids at the cartilage surface [23]. Boundary lubrication appears to depend on the physical characteristics of hyaluronan and not on its presence alone, since the kinetic friction coefficient of synovial fluid rises after joint injury in association with reductions in hyaluronan concentration and molecular weight, and the addition of high-molecular-weight hyaluronan lowers the friction coefficient toward values measured in uninjured fluid [24]. These observations indicate that the molecular state of hyaluronan, including its molecular weight, concentration, and network organization, contributes to tribological function, which provides a rationale for the hypothesis that networked or chemically modified hyaluronan may sustain boundary lubrication over a longer period than an unmodified solution. Direct tribometric comparisons of the hexadecylamide derivative and BDDE cross-linked gels against linear hyaluronan are limited, however, and this inference remains to be confirmed experimentally.

### 5.4. Resistance to Enzymatic and Free-Radical Degradation

Native hyaluronan is degraded enzymatically by hyaluronidase and is depolymerized by reactive oxygen species, processes that shorten its residence within tissues and fluids [1]. Mechanical loading of chondrocytes generates reactive oxygen species and depolymerizes hyaluronan, an effect attenuated by superoxide dismutase, which links mechanical stress to oxidative chain scission [25]. Covalent cross-linking with BDDE increases resistance to enzymatic degradation and prolongs the persistence of the network, with the degree of resistance dependent on cross-linking density and conditions [13,18]. A direct analytical comparison across the three states is also available, and it separates two effects that are easily conflated. Unmodified high-molecular-weight hyaluronan, the hexadecyl amide derivative, a BDDE network and a divinyl sulfone network were each formulated at 8 mg/mL and incubated with bacterial collagenase as a model of the human matrix metalloproteinases. After two hours at 37 °C, residual enzyme activity was 81.3 ± 5.5% with unmodified hyaluronan, 8.3 ± 0.3% with the BDDE network, 12.5 ± 0.8% with the divinyl sulfone network and 7.8 ± 0.2% with the hexadecyl amide derivative [26]. When the same polymers were thermally depolymerized to remove the contribution of viscosity and physical entrapment, the effect of both cross-linked networks was largely abolished, residual activity returning to 81.7 ± 5.8% and 74.3 ± 3.7%, whereas the effect of the derivative persisted and increased, residual activity falling to 2.3 ± 0.1% [26]. The protection conferred by the covalent networks in that assay is therefore substantially physical and arises from the network itself, while the derivative acts in addition as a competitive enzyme inhibitor through its alkyl chain. Screening against ten human matrix metalloproteinases showed selectivity for MMP-8 and MMP-13, with an inhibition constant for human MMP-13 of 61.7 ± 7.0 μM in buffer and 106.1 ± 9.2 μM measured ex vivo in synovial fluid, and incubation in osteoarthritic synovial fluid preserved the molecular weight of endogenous hyaluronan against hyaluronidase-2 [26]. Two limits apply to these data: they measure inhibition of enzyme activity rather than resistance of the material to degradation, which are distinct properties, and the assays required thermal depolymerization, so the relation to the intact injected product is indirect. Complementary characterization that would document network integrity by other routes, in particular equilibrium swelling ratio, differential scanning calorimetry and thermogravimetric analysis, has not been reported for these viscosupplement formulations in the literature retrieved here, and Table 2 is restricted accordingly to properties for which measurements exist. The physical network of the hexadecylamide derivative resists dispersion more effectively than the unmodified solution, consistent with the longer intra-articular residence reported for the derivative [12,27]. The comparative physicochemical properties of the three states are collected in Table 2.

**Table 2:** Comparative physicochemical and rheological characteristics of the three hyaluronan material states.

Property	Linear Hyaluronan	HYADD-4 (Hexadecylamide)	BDDE Cross-Linked Hyaluronan	Measurement Basis
Predominant behavior	Viscous solution	Transient physical network	Elastic, solid-like gel	Oscillatory rheology of marketed products at 25 °C, 0.5–3 Hz [12]
Storage vs. loss modulus	Loss dominates across the knee band	Storage exceeds loss across the knee band; crossover below the band	Storage exceeds loss across the range	Frequency sweeps against marketed comparators [12]
Determinant of viscosity	Molecular weight and concentration	Hydrophobic association of C16 side chains	Cross-link density	[1,12,13]
Collagenase activity in the presence of the material (8 mg/mL, 2 h, 37 °C)	81.3 ± 5.5% residual	7.8 ± 0.2% residual, falling to 2.3 ± 0.1% after depolymerization	8.3 ± 0.3% residual, returning to 81.7 ± 5.8% after depolymerization	Collagenase assay as a model of human MMPs [26]
Free-radical depolymerization	Susceptible	Not measured directly for this derivative	Reduced for cross-linked hylan	[1,25]; no direct measurement retrieved for HYADD-4
Reported intra-articular residence	Short	Prolonged relative to solution	Prolonged	[12,13,27]

Each entry is referenced to the measurement that supports it. Where no measurement was retrieved for a given state, this is stated rather than inferred. Absolute values depend on the molecular weight, concentration, and cross-linking conditions of the specific preparation, and the collagenase data refer to inhibition of enzyme activity rather than to degradation of the material itself. MMP, matrix metalloproteinase.

### 5.5. Methods for Characterizing Network State, and What Each One Establishes

The criteria set out in Section Criteria Applied in This Review for Treating a Hyaluronan Preparation as a Gel are not addressed by every technique that appears in the hydrogel literature, and the distinction matters for materials of this kind. Oscillatory rheology in the linear viscoelastic regime addresses both criteria directly, because the frequency dependence of G′ and G″ reports on the presence and the persistence of junctions. Dynamic light scattering and fluorescence recovery after photobleaching address network architecture, returning a correlation length and a diffusive mesh size; for the hexadecylamide derivative these give 24 nm at 3 mg/mL falling to 13 nm at 8 mg/mL, relaxation times of about 210 to 85 microseconds across the same range, and a sieving effect on dextran probes of 250 and 500 kDa but not on 20 kDa [12,15]. Enzymatic assays address persistence rather than structure, and the thermal depolymerization control described in Section 5.4 is what separates a physical obstruction from a genuine inhibitory interaction [26].

Equilibrium swelling is a legitimate network measurement, related to cross-link density through the Flory–Rehner treatment, but its application to these materials carries two conditions that are often left unstated. It is defined for a pre-formed network placed in excess solvent, which describes a covalently cross-linked gel but not a physically associated one, since dilution shifts the association equilibrium of the hexadecylamide derivative toward the dissociated state, and the network the measurement is meant to probe does not survive the measurement. It is also strongly medium-dependent. In BDDE cross-linked hyaluronan prepared by two different mixing routes, swelling ratios differed significantly between preparations in distilled water yet showed no significant difference in phosphate-buffered saline, while the same preparations differed in resistance to hyaluronidase and in pore size on scanning electron microscopy [28]. Swelling of hyaluronan nanogels cross-linked with BDDE, divinyl sulfone or poly(ethylene glycol) bis(amine) is likewise pH-dependent, following ionization of the carboxyl groups [29], and comparisons among marketed fillers report lower swelling for poly(ethylene glycol) cross-linked than for BDDE cross-linked material [30]. Swelling therefore discriminates between cross-linking chemistries under controlled laboratory conditions, and discriminates poorly at the ionic strength relevant to intra-articular use.

Thermal methods occupy a different position again. Differential scanning calorimetry and thermogravimetric analysis report transitions and mass loss on heating, and in the hyaluronan literature they are applied mainly to dried films and composite matrices, where they characterize the polymer and any added component rather than the hydrated network [31]. Neither method distinguishes a percolated network from an entangled solution of the same composition, which is the distinction at issue here, and in a preparation that is more than ninety-nine percent water by mass a thermogravimetric trace is dominated by evaporation of that water. A comparable limitation applies to probes based on water mobility: across BDDE cross-linked hyaluronan hydrogels of differing gel content, swelling ability and elastic behavior, the diffusion coefficient of water protons did not differ between hydrogels, and only transverse relaxation, which is governed by chemical exchange, varied appreciably among them [32]. Swelling, calorimetry and thermogravimetry are therefore reported here for what they establish about network density and thermal behavior, and are not treated as evidence of gel state.

Two gaps follow from this survey, and are stated rather than filled. Equilibrium swelling, differential scanning calorimetry and thermogravimetric analysis have been reported for laboratory preparations of cross-linked hyaluronan and for composite matrices, but not, in the literature retrieved here, for the marketed intra-articular formulations discussed in Section 7 and Section 8, which are supplied hydrated at a fixed concentration. No formal Winter–Chambon analysis has been published for the hexadecylamide derivative. Table 2 is accordingly restricted to properties for which measurements on these materials exist, and both gaps are carried into Section 10 and Section 11.

## 6. Biological Mechanism of Action

Intra-articular hyaluronan is generally held to act through two complementary mechanisms, one biomechanical and one biological [33]. One distinction governs this section throughout. Part of the evidence summarized below was obtained with unmodified polymer of defined molecular weight and concerns hyaluronan as a class; it says nothing directly about network state, and extending it to the modified materials is an extrapolation. The remainder was obtained with the hexadecylamide derivative or with covalently cross-linked material and is attributable to those materials specifically. The two are kept separate below and are marked as such in Figure 4. The biomechanical contribution arises from the viscoelasticity and lubrication that the material confers on synovial fluid, while the biological contribution arises from interactions between hyaluronan and cell-surface receptors on synoviocytes and chondrocytes that modulate gene expression and inflammation [33]. A systematic review of the preclinical literature identified chondroprotection as the most frequently reported effect, followed by stimulation of proteoglycan and glycosaminoglycan synthesis, anti-inflammatory activity, and mechanical and analgesic actions, with binding to the CD44 receptor cited most often as the underlying biological mechanism and with high-molecular-weight preparations reported as more effective than low-molecular-weight ones [4]. That body of work concerns hyaluronan as a class and is not resolved by network state.

The biological activity of hyaluronan depends on its molecular weight. High-molecular-weight hyaluronan tends to exert anti-inflammatory effects, whereas low-molecular-weight fragments can act as pro-inflammatory signals through interactions with CD44, toll-like receptors, and other receptors that converge on nuclear factor kappa B signaling [34]. This molecular-weight dependence connects the physical state of the material to its biological consequences, since processes that depolymerize the polymer can shift its activity from protective toward inflammatory [34]. This framework is defined by chain length and has been demonstrated for hyaluronan as a class. It does not establish that network architecture as such determines receptor signaling, and no study identified here compares receptor engagement across the three material states at matched molecular weight.

Comparative studies of the hexadecylamide derivative and unmodified hyaluronan in human osteoarthritic cells indicate that the derivative more effectively attenuates interleukin-1-beta-induced expression of matrix-degrading enzymes, including matrix metalloproteinase-1 and matrix metalloproteinase-13 and the aggrecanase ADAMTS5, and of inflammatory mediators such as interleukin-6, with greater inhibition of the associated signaling cascades [35]. The proliferative response of chondrocytes to both materials is abolished by an anti-CD44 antibody, which indicates that the effect is mediated through that receptor, and the derivative additionally promotes expression of the anti-inflammatory cytokine interleukin-10 while showing longer joint residence and higher viscoelasticity than the parent polymer [27]. In an ovine meniscectomy model, both unmodified hyaluronan and the hexadecylamide derivative reduced synovial pathology, and the derivative reduced vascularity and intimal hyperplasia while increasing synthesis of high-molecular-weight hyaluronan by synovial fibroblasts [36]. A companion gait analysis in the same model showed partial recovery of ground reaction forces after treatment with either preparation [37]. The receptor interactions and molecular-weight-dependent signaling described here are represented in Figure 4.

**Figure 4:**
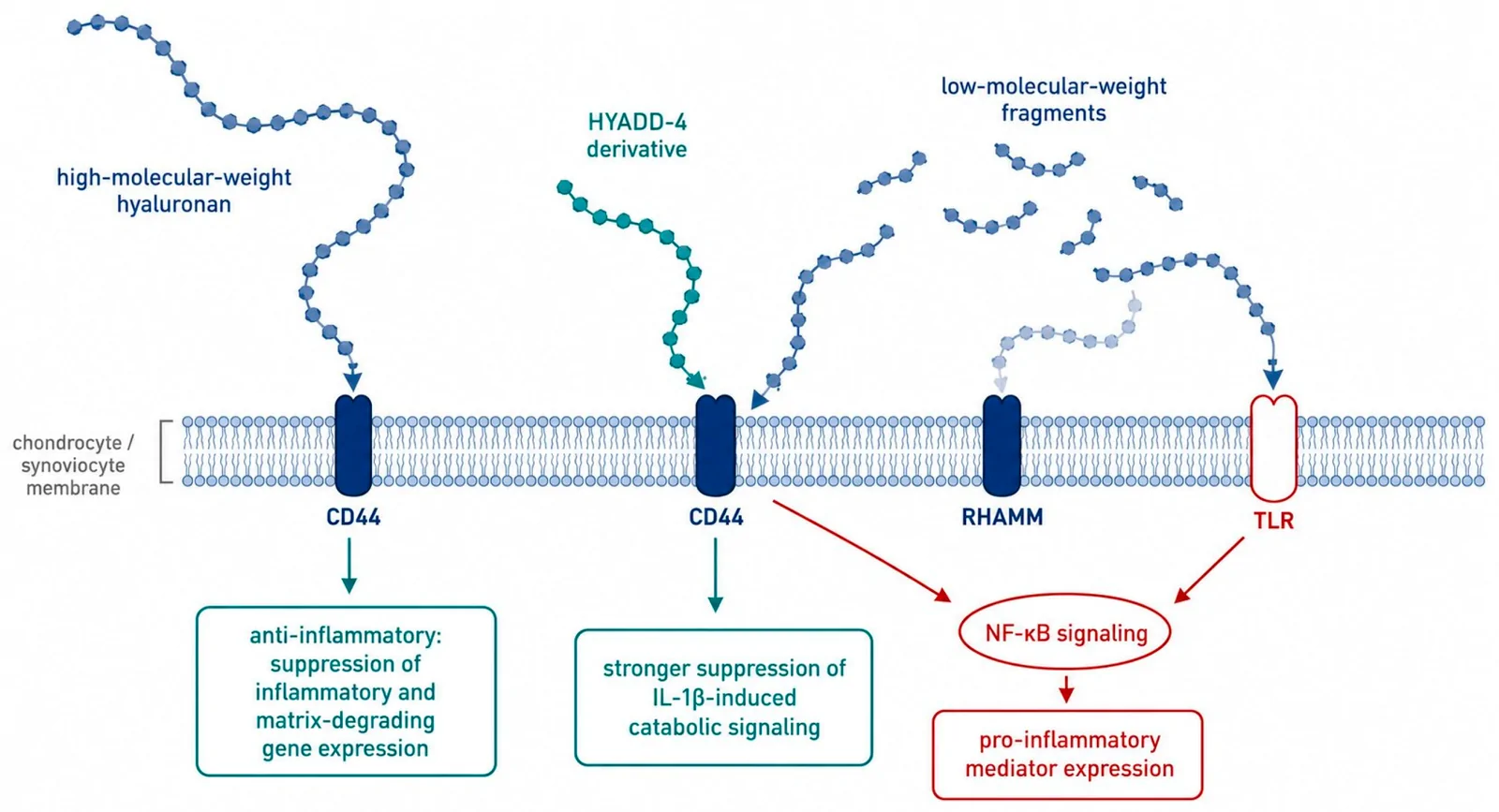
Receptor-mediated mechanisms of hyaluronan in the joint, distinguishing relationships demonstrated for hyaluronan as a class from those demonstrated for the modified material. Demonstrated for hyaluronan generally, and therefore extrapolated when applied to the modified materials [4,34]: high-molecular-weight hyaluronan engages CD44 on chondrocytes and synoviocytes and is associated with suppression of inflammatory and matrix-degrading gene expression, whereas low-molecular-weight fragments engage CD44 and toll-like receptors and promote nuclear factor kappa B signaling and pro-inflammatory mediator expression. Demonstrated specifically for the hexadecylamide derivative in direct comparison with the unmodified polymer [27,35]: stronger suppression of interleukin-1-beta-induced catabolic signaling and of matrix-degrading enzyme expression, CD44-dependent chondrocyte proliferation abolished by an anti-CD44 antibody, and induction of interleukin-10. The figure is a schematic summary of published relationships and carries no quantitative data. Color key: blue chains represent hyaluronan, both the high-molecular-weight polymer and low-molecular-weight fragments, and the teal chain represents the hexadecylamide derivative; dark blue shapes represent the CD44 and RHAMM receptors, and the red outline represents the toll-like receptor; teal boxes and arrows indicate anti-inflammatory and anti-catabolic outcomes, whereas red arrows, ellipse, and box indicate the pro-inflammatory pathway. The light, translucent chain directed to RHAMM is shown with lower emphasis because the contribution of RHAMM in chondrocytes and synoviocytes is less well characterized than that of CD44 and toll-like receptors.

## 7. Networked Versus Conventional Linear Hyaluronan

The preceding sections support a structural and functional distinction between conventional linear hyaluronan and the networked materials. The unmodified polymer provides lubrication and viscoelastic support but is cleared and degraded relatively quickly, which is consistent with the multiple-injection regimens historically used for solution-based viscosupplements [1,4,38]. The networked materials, whether physically associated or covalently cross-linked, exhibit higher viscoelasticity and greater resistance to degradation, which provides a mechanistic rationale for longer residence [12,13]. The link from that rationale to the number of injections should be made carefully. Injection schedules are properties of individual products and follow from dose, concentration, formulation, trial protocol, and regulatory labeling as much as from network architecture. A smaller number of injections is therefore not a general consequence of being a physical or a covalent network, and the regimens given in Table 3 are those of the specific products cited in this review rather than a property of the material state.

This rationale should be stated carefully. The evidence that the hexadecylamide derivative offers biological and mechanical advantages over unmodified hyaluronan derives principally from in vitro studies on human osteoarthritic cells and from large-animal models [27,35,36,37], together with the regulatory dosing of the marketed product [10]. A direct, peer-reviewed, head-to-head randomized controlled trial comparing the hexadecylamide derivative with a conventional linear hyaluronan was not identified in the indexed literature, and the rationale for a reduced number of injections therefore rests on rheological and preclinical data and on product labeling rather than on a published clinical comparison. The principal points of contrast among the three states are summarized in Table 3.

**Table 3:** Comparison of conventional linear hyaluronan with the physically associated and covalently cross-linked networks.

Attribute	Linear Hyaluronan	HYADD-4 (Hexadecylamide)	BDDE Cross-Linked Hyaluronan
Structure	Entangled solution	Reversible physical network	Covalent network
Representative products cited here	Hyalubrix (1500 kDa, 15 mg/mL) [12]	Hymovis (HYADD-4, 8 mg/mL) [10,12]	HYA-JOINT Plus (20 mg/mL) [39,40]; Durolane, NASHA (20 mg/mL) [12,40]; Crespine Gel [41]
Durability in joint	Short	Prolonged relative to solution	Prolonged
Labeled injection regimen of the products above	Multiple injections	Two injections one week apart [10,42]	Single injection [39,40,41]
Rheological profile	Viscous-dominated across the knee band	Storage modulus above loss modulus across 0.5–3 Hz	Elastic, solid-like
Strength of comparative clinical evidence	Long-standing use	Preclinical and rheological; no head-to-head randomized trial against linear hyaluronan identified	Randomized single-injection trials against other hyaluronan products, not against linear solutions

Injection regimens are those of the named products and reflect product labeling and trial protocols, not a property of the material state; the comparison is qualitative. Cross-linking chemistry differs among the products grouped in the third column and is identified per product in Section 8.1 where the source reports it. NASHA: non-animal stabilized hyaluronic acid.

## 8. Clinical Evidence and Regenerative Applications

### 8.1. Osteoarthritis and Viscosupplementation

Clinical experience with the hexadecylamide derivative in knee osteoarthritis includes a retrospective series in which two intra-articular injections administered one week apart were associated with reductions in the Western Ontario and McMaster Universities Osteoarthritis Index and in analgesic use over six months, with relief of resting pain reported in a subgroup for approximately twelve months [42]. A single-center, single-blind randomized controlled trial comparing the derivative with methylprednisolone acetate reported better function with the derivative through twenty-six weeks and no significant difference at one year [43]. A multicenter open-label follow-up study reported sustained benefit over a longer period [44], and a prospective pilot study in professional soccer players with knee chondropathy reported improvement in clinical scores over six months [45]. A randomized controlled study of the derivative in degenerative meniscal tears reported reductions in pain and improvements in function together with magnetic resonance imaging changes in the lesion [46]. In an open-label, multicenter pilot study in 46 patients, designed by its authors to identify candidate biomarkers for a subsequent placebo-controlled trial rather than to demonstrate efficacy, the derivative was associated with increased type II collagen turnover and with an increase in cartilage volume and thickness in the lateral femoral and lateral trochlear compartments on magnetic resonance imaging, with no such change in the medial compartment, alongside a reduction in an effusion score [47]. These imaging and biochemical findings are exploratory. They were obtained without a control group, and they should be read as supportive signals rather than as evidence of cartilage regeneration; the distinction between symptomatic improvement and structural repair is not resolved by these data. No randomized trial comparing the hexadecylamide derivative directly with a conventional linear hyaluronan was identified in the indexed literature, and the comparative clinical benefit of the derivative over the linear solution therefore remains untested.

Preclinical work supports a chondroprotective effect and a capacity to act as a carrier for growth factors. In a rabbit anterior cruciate ligament transection model, the derivative delayed cartilage degeneration, and its combination with recombinant bone morphogenetic proteins produced effects greater than either component alone [48]. Not all data are positive, however. In a non-invasive murine tibial-loading model, intra-articular delivery of the derivative with or without platelet-rich plasma did not protect cartilage or synovium, with only minor trends observed in bone, which indicates that the benefit is context-dependent [49].

For covalently cross-linked products, randomized controlled trials have examined single-injection regimens. The cross-linking chemistry differs among these products and is not always stated in the clinical reports, so the properties described for BDDE networks in Section 4.3 should not be transferred to cross-linked hyaluronan as a class. A level-I double-blind trial in 132 patients compared a single injection of HYA-JOINT Plus, a cross-linked hyaluronan at 20 mg/mL whose cross-linking chemistry is not specified in the report, with a single injection of Synvisc-One, which is hylan G-F 20, a mixture of high-molecular-weight hylan A and cross-linked hylan B; the cross-linked product reduced the visual analog pain score more than the comparator at one, three, and six months [12,39]. A fifty-two-week double-blind randomized trial in 142 patients compared the same cross-linked product with Durolane, a non-animal stabilized hyaluronic acid, and reported that both were effective over the follow-up period, with one superior over part of the interval [40]. A single-arm post-marketing phase IV study of Crespine Gel, a cross-linked hyaluronan of unstated chemistry, reported benefit extending to nine months in 84 evaluable patients and included an author affiliated with the distributor [41]. These trials support the durability associated with covalent networks, but they compare cross-linked products with one another rather than with unmodified solutions, and the comparator in each case is itself a modified material.

### 8.2. Hyaluronan Scaffolds and Carriers in Regenerative Procedures

Beyond viscosupplementation, hyaluronan serves as a scaffold and carrier in cell-based cartilage repair. A hyaluronan-based scaffold combined with activated bone marrow aspirate concentrate was reported to provide more durable cartilage repair than microfracture at five years in a cohort of physically active patients [50], and prospective follow-up of the same one-stage technique reported good to excellent outcomes at a mean of eight years across a range of lesion sizes [51] and maintenance of clinical improvement at a mean of fourteen years [52]. These findings position the hyaluronan scaffold as a carrier that supports cells over an extended period.

The design of hyaluronan hydrogels for cartilage tissue engineering has been reviewed in detail, with attention to the tuning of nutrient diffusion, matrix deposition, degradation, and the delivery of cells and growth factors within the network [53]. Contemporary work continues to develop hyaluronan-containing injectable hydrogels as carriers for cells and biological agents, including a chitosan-hyaluronan hydrogel delivering mesenchymal stem cells together with chondrocyte-derived extracellular vesicles that supported cartilage regeneration in an animal model [54]. The clinical studies discussed in this section are summarized in Table 4 and the preclinical studies in Table 5.

**Table 4:** Clinical studies of modified and cross-linked hyaluronan discussed in this review.

Study	Material and Regimen	Design, Comparator and Number of Patients	Follow-up and Primary Endpoint	Principal Outcome	Safety and Sponsorship
Priano 2017 [42]	HYADD-4, 24 mg/3 mL, two injections one week apart	Retrospective case series; no comparator; 937 enrolled, 698 evaluable	6 months, with pain followed to 12 months in a subgroup of 106; no prespecified primary endpoint	WOMAC reduced by 56.3%; use of NSAIDs or acetaminophen at least twice weekly fell from 48.8% to 19.6%	Adverse events in 11.2%, mainly arthralgia; no allergic or systemic reactions; sponsorship not stated
Bisicchia 2016 [43]	HYADD-4, two injections	Single-centre, single-blind randomized; comparator methylprednisolone acetate; 75 per group	52 weeks; primary endpoint WOMAC at 26 weeks	Better WOMAC with the derivative at 26 weeks; no significant difference at 52 weeks	Mild adverse effects of similar incidence in both arms; first author a consultant for the manufacturer; sponsorship not stated
Zorzi 2016 [46]	HYADD-4 for degenerative meniscal tears	Single-site, observer-blind, randomized parallel-group; 50 patients	60 days; outcomes VAS pain, WOMAC and MRI lesion size	Reduced VAS pain from day 14; reduction in lesion length and depth on MRI versus control	Local tolerability reported as good; one author affiliated with the manufacturer
Henrotin 2019 [47]	HYADD-4, 8 mg/mL, 3 mL; two cycles six months apart, each of two injections one week apart	Open-label, prospective, multicentre pilot; no control group; 46 patients	12 months; primary endpoint change in type II collagen biomarkers	Increase in Coll2-1 and PIIANP; cartilage volume and thickness increased in lateral compartments only; effusion score reduced; KOOS improved	Low incidence of injection-site pain; two authors affiliated with the manufacturer; explicitly designed as a pilot for a later controlled trial
Sun 2017 [39]	HYA-JOINT Plus, cross-linked, 20 mg/mL, single 3 mL injection	Randomized, double-blind, level I; comparator Synvisc-One (hylan G-F 20), 8 mg/mL, 6 mL; 132 randomized, 121 analysed	6 months; primary endpoint change in VAS pain at 6 months	Greater VAS reduction than comparator at 1, 3 and 6 months; WOMAC stiffness favoured the cross-linked product at 6 months	No serious adverse events; sponsorship not stated
Ko 2022 [40]	HYA-JOINT Plus, single 3 mL injection	Randomized, double-blind; comparator Durolane (NASHA); 142 randomized	52 weeks; primary endpoint VAS pain at 26 weeks	Lower VAS pain than comparator at 26 weeks, maintained to 39 weeks; both products effective to 52 weeks	Mild and self-limited adverse events in 40.8%; sponsorship not stated
Bashaireh 2015 [41]	Crespine Gel, cross-linked, single injection	Post-marketing phase IV, single arm, no comparator; 109 recruited, 84 analysed	9 months; WOMAC	Peak improvement at 5 months, with significant improvement retained at 9 months	Local and transient reactions; one author affiliated with the distributor
Gobbi 2016 [50]	Hyaluronan-based scaffold with activated bone marrow aspirate concentrate	Cohort study, level 2; allocation by insurance coverage rather than randomization; comparator microfracture; 50 patients	5 years; IKDC objective and subjective, Lysholm, Tegner and KOOS	IKDC objective normal or nearly normal in all scaffold patients at 5 years versus 28% after microfracture	Safety not reported in detail; non-random allocation limits comparability
Whyte 2024 [52]	Hyaluronan-based scaffold with bone marrow aspirate concentrate	Prospective case series, level 4; no comparator; 26 patients, 22 analysed	Mean 14.0 years; VAS and KOOS	Median VAS fell from 5.0 to 0.6; all KOOS subscales improved	Three treatment failures and one later unicompartmental arthroplasty

Sponsorship is reported where the source publication states it, either as a funding declaration or through an author affiliation with the manufacturer or distributor; “not stated” means that no such information was given in the publication retrieved. IKDC, International Knee Documentation Committee; KOOS, Knee injury and Osteoarthritis Outcome Score; MRI, magnetic resonance imaging; NASHA, non-animal stabilized hyaluronic acid; NSAID, non-steroidal anti-inflammatory drug; PIIANP, procollagen type II N-terminal propeptide; VAS, visual analogue scale; WOMAC, Western Ontario and McMaster Universities Osteoarthritis Index.

**Table 5:** Preclinical and in vitro studies of modified and cross-linked hyaluronan discussed in this review.

Study	Material	Model	Principal Outcome
Smith 2013 [35]	HYADD-4 versus unmodified hyaluronan	Human osteoarthritic chondrocytes and synoviocytes in vitro	Greater attenuation of interleukin-1-beta-induced MMP-1, MMP-13 and ADAMTS5 and of interleukin-6
Brun 2012 [27]	HYADD-4 versus unmodified hyaluronan	Human osteoarthritic chondrocytes and fibroblast-like synoviocytes in vitro	CD44-dependent proliferation abolished by anti-CD44 antibody; induction of interleukin-10
Smith 2008 [36]	HYADD-4 and unmodified hyaluronan	Ovine meniscectomy	Both reduced synovial pathology; the derivative reduced vascularity and intimal hyperplasia and increased synthesis of high-molecular-weight hyaluronan
Cake 2008 [37]	HYADD-4 and unmodified hyaluronan	Ovine meniscectomy with gait analysis	Partial recovery of ground reaction forces with either preparation
Mainil-Varlet 2013 [48]	HYADD-4	Rabbit anterior cruciate ligament transection	Delayed cartilage degeneration; greater effect combined with recombinant bone morphogenetic proteins than either component alone
Duan 2017 [49]	HYADD-4 with or without platelet-rich plasma	Non-invasive murine tibial loading	No protection of cartilage or synovium; only minor trends in bone
Pavan 2016 [26]	HYADD-4, BDDE and divinyl sulfone networks, unmodified hyaluronan	Collagenase and hyaluronidase assays, human MMP panel, ex vivo synovial fluid	Derivative inhibits MMP-8, MMP-13 and hyaluronidase-2; apparent protection by cross-linked networks is lost after depolymerization

ADAMTS5, a disintegrin and metalloproteinase with thrombospondin motifs 5; BDDE, 1,4-butanediol diglycidyl ether; MMP, matrix metalloproteinase.

## 9. Synthesis: What the Evidence Supports Across the Three States

Read together, the chemistry, the rheology, the degradation data and the clinical record do not place the three states on a single scale of increasing performance. They place them on three different footings, each supported by a different kind of evidence. For native linear hyaluronan the evidence is clinical and long-standing but structurally unremarkable: the material is an entangled solution, its intra-articular residence is short, and the multiple-injection regimens historically used follow from that. For the covalently cross-linked network the structural evidence is the strongest of the three, since the network satisfies both the percolation and the mechanical criterion, and the clinical evidence comes from randomized single-injection trials; the qualification is that those trials compare cross-linked products with one another rather than with an unmodified solution, so they establish relative and not absolute benefit [39,40,41]. For the hexadecylamide derivative the position is the reverse of what its clinical positioning would suggest. The mechanistic and biochemical evidence is the most specific of the three, including CD44-dependent effects demonstrated against the unmodified polymer [27,35] and direct enzyme inhibition with measured inhibition constants [26], while the structural claim is the weakest, because the formal gel criterion has not been tested.

This asymmetry accounts for a pattern that is otherwise puzzling in the literature. Comparative clinical trials of these materials rarely separate them by network state, because the products differ simultaneously in concentration, molecular weight, cross-linking chemistry and injection schedule, and because the comparator is itself usually a modified material. The rheological literature, by contrast, separates them cleanly, and shows the separation to be large: at 8 mg/mL the derivative is elastic-dominated across the entire walking-to-running band, while the parent polymer at the same concentration is not [12]. The distance between a clear physical distinction and an unresolved clinical one is the central open problem in this field, and none of the studies reviewed here closes it.

A second point follows for the interpretation of mechanism. The receptor biology most often invoked to explain the action of intra-articular hyaluronan is organized by molecular weight, not by network state [4,34]. Network state governs residence, and residence governs how long a given molecular weight remains present in the joint, so the two are connected. No study identified here, however, compares receptor engagement across the three states at matched molecular weight, and until that comparison is made the biological argument for network modification remains indirect. The distinction defended in this review is therefore physical and, where the data allow, biochemical. It is not yet a demonstrated clinical distinction, and no such claim is made.

## 10. Limitations and Future Perspectives

Several limitations qualify the evidence reviewed here. The literature specific to the hexadecylamide derivative is limited in volume and heterogeneous in design, and it includes retrospective series and open-label studies alongside randomized trials. Head-to-head randomized comparisons between the modified materials and conventional linear hyaluronan were not identified. The comparators in the randomized trials of cross-linked products are themselves modified materials, so those trials establish relative rather than absolute benefit. Manufacturer or distributor involvement is recorded in Table 4 for the studies whose publications state it, and it extends to the pilot study that provides the imaging and biomarker signals [41,43,46,47]; those signals are exploratory and uncontrolled. Direct tribological measurements comparing the three states are limited, so the link between boundary-lubrication principles and the modified materials remains an inference rather than a demonstrated property.

The three-state framework proposed here is intended as an organizing distinction rather than a definitive taxonomy, and its boundaries are not always sharp. Hybrid materials that combine physical and covalent junctions, such as pre-cross-linked microgels stabilized by additional electrostatic interactions, occupy intermediate positions and offer injectable, shear-thinning behavior [55]. Stimuli-responsive hyaluronan and bioinspired hydrogel systems point toward materials whose properties can be adjusted in situ and whose networks can be designed for controlled release and cell culture [56]. Direct comparative studies of rheology, tribology, degradation, and clinical outcome across defined material states would strengthen the rational basis for material selection.

## 11. Conclusions

The limitations of this review follow from its scope and are stated here rather than left to a separate section. This is a narrative review and generates no primary data, so every quantitative statement is bounded by what the cited studies measured and by the conditions under which they measured it. The literature specific to the hexadecylamide derivative is small, heterogeneous in design, and in several cases supported by the manufacturer, as recorded in Table 4. Characterization that would document network integrity by routes other than rheology, in particular equilibrium swelling, differential scanning calorimetry and thermogravimetric analysis, has not been reported for the marketed intra-articular formulations, and the formal rheological test of the gel state has not been performed for the derivative (Section 5.5). Direct tribological comparison across the three states is likewise absent. The three-state framework is therefore proposed as an organizing distinction that is well supported chemically and rheologically, partly supported biochemically, and not yet tested clinically.

Hyaluronan-based materials used in orthopaedic and regenerative medicine span at least three distinct material states: a viscous linear solution, a physically associated network formed by the hexadecylamide derivative, and a covalent network formed by cross-linking with BDDE. These states differ in their network chemistry, their rheological and tribological behavior, their resistance to enzymatic and oxidative degradation, and the strength and type of evidence supporting their clinical use. Treating them as a single category obscures these differences. Recognizing the material state to which a given preparation belongs clarifies the interpretation of comparative data and supports a more rational selection of materials. The framework is bounded by the evidence available. Only the covalent network satisfies the formal gel criterion across the range examined; the hexadecylamide derivative is elastic-dominated across the working frequencies of the knee but lacks a published Winter–Chambon analysis and is therefore treated here as a transient physical network, and the statement that linear hyaluronan is not a gel is restricted to the formulations used in marketed viscosupplements. Complementary characterization of network integrity, direct tribological comparison across states, and a randomized comparison of a modified material against a conventional linear solution are the measurements that would test the framework, and each remains to be done.

## Figures and Tables

**Figure 1:**
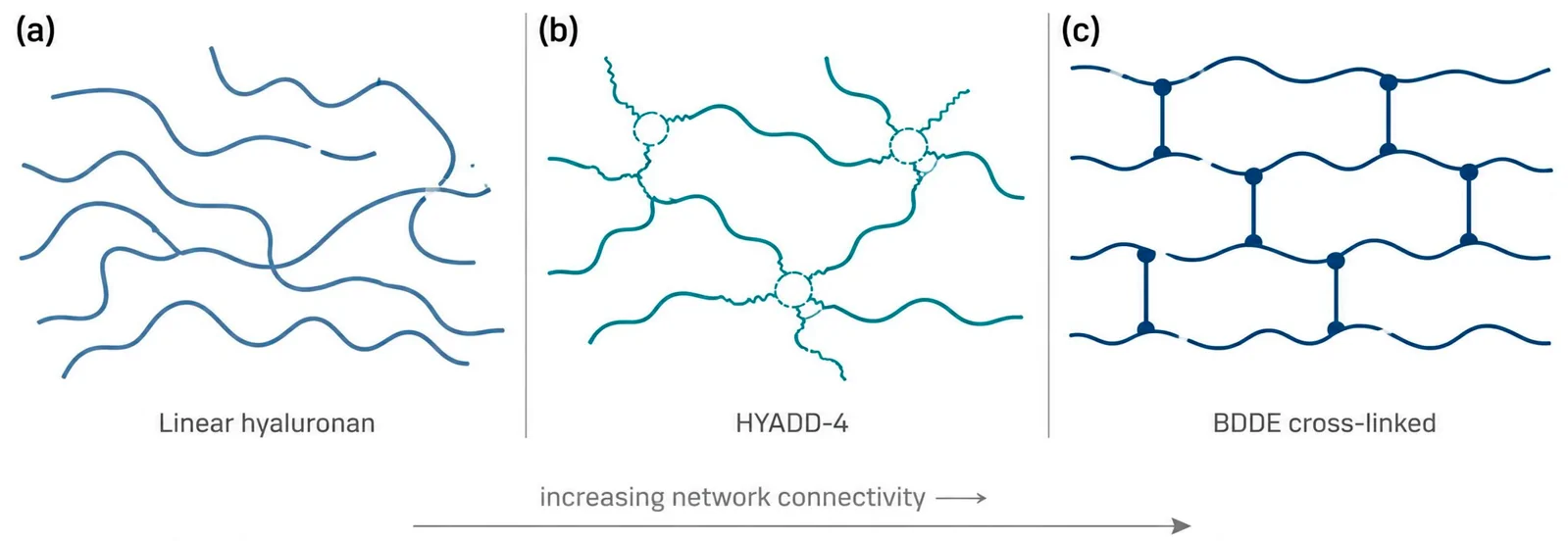
Schematic representation of the three material states of hyaluronan. (**a**) Native linear hyaluronan in solution, shown as entangled, non-junctioned chains forming a viscous solution; (**b**) the hexadecylamide derivative (HYADD-4), shown as chains joined by reversible hydrophobic associations of C16 side chains, forming a physical network; (**c**) BDDE cross-linked hyaluronan, shown as chains joined by covalent ether bonds, forming a three-dimensional covalent network.

**Figure 2:**
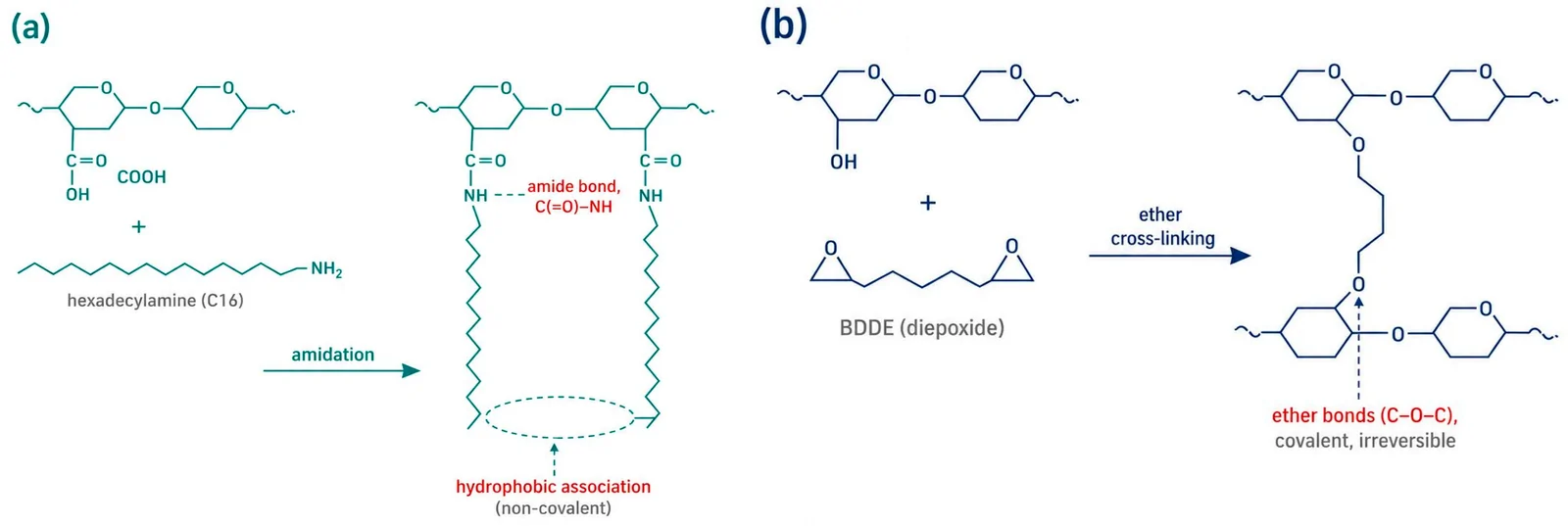
Network-forming chemistry of the two modified hyaluronan states. (**a**) Amidation of hyaluronan carboxyl groups with hexadecylamine introduces C16 side chains that associate through hydrophobic interactions to form a reversible physical network (HYADD-4); (**b**) reaction of hyaluronan hydroxyl groups with BDDE under alkaline conditions forms covalent ether linkages that constitute an irreversible three-dimensional network.

**Figure 3:**
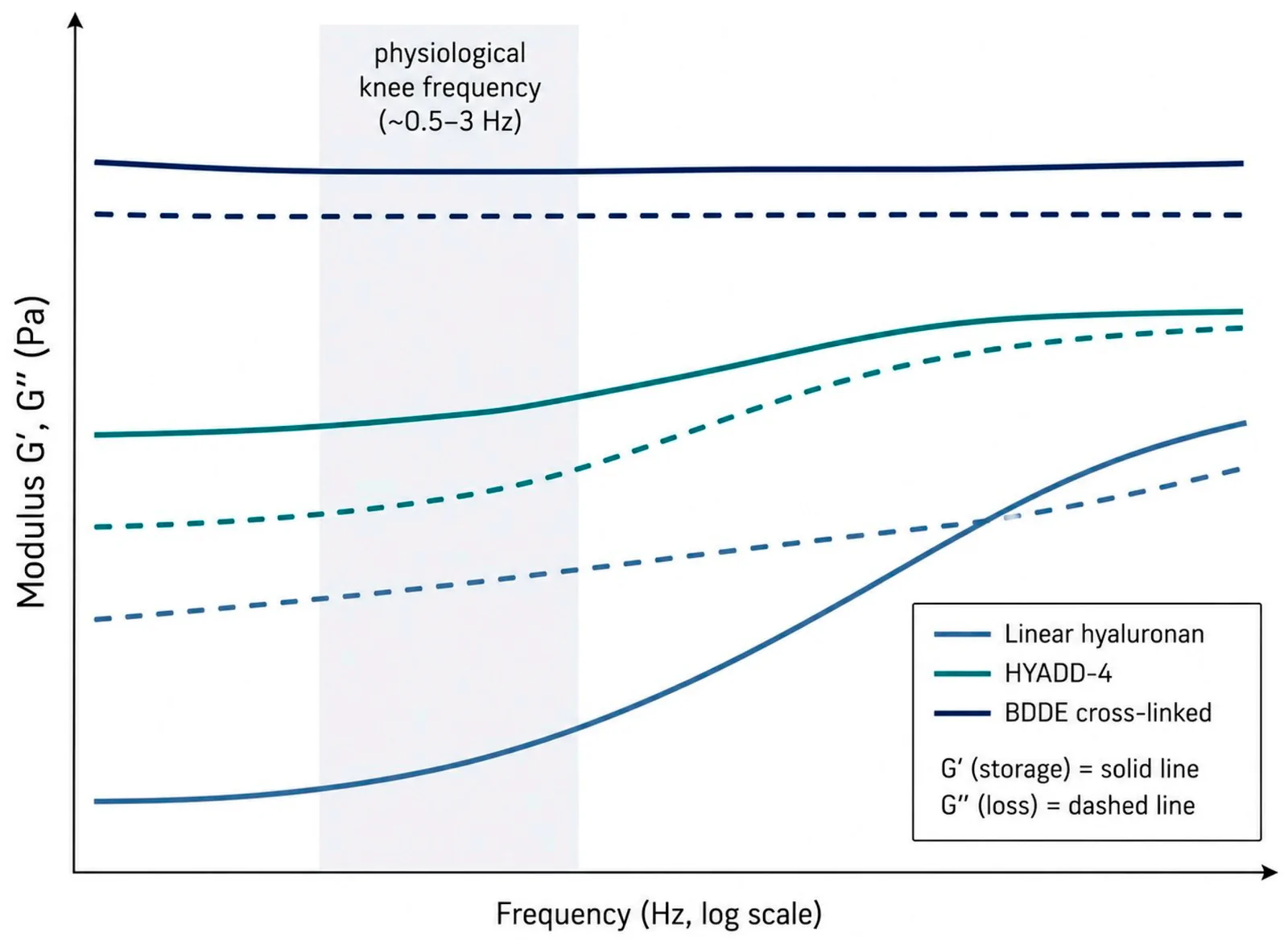
Schematic oscillatory rheological behavior of the three material states, showing the storage modulus and loss modulus as a function of frequency. The curves are schematic: they are not experimental data, the axes are illustrative and not drawn to scale, and no quantitative value should be read from them. The linear solution is dominated by the loss modulus over the physiological band; the physical network of the hexadecylamide derivative is dominated by the storage modulus across the walking-to-running band of the knee, with its crossover lying below that band; the covalent network shows storage modulus exceeding loss modulus across the range. The crossover of the two moduli is shown as an approximate practical marker of the transition and not as the formal gel criterion, which is the frequency independence of tan δ discussed in Section 5.2.

**Table 1:** Defining features of the three hyaluronan material states.

Feature	Linear Hyaluronan	HYADD-4 (Hexadecylamide)	BDDE Cross-Linked Hyaluronan
Physical form	Viscoelastic solution	Physical hydrogel	Covalent hydrogel
Network type	None (entanglement only)	Non-covalent, reversible	Covalent, irreversible
Junction chemistry	Chain entanglement	Hydrophobic C16 association; amide-linked side chains	Ether bonds via BDDE
Representative use	Viscosupplement; lubricant	Intra-articular viscosupplement	Dermal filler; cross-linked viscosupplement

HYADD-4, hexadecylamide derivative of hyaluronan; BDDE, 1,4-butanediol diglycidyl ether; C16, hexadecyl chain.

## Data Availability

No new data were created or analyzed in this study. Data sharing is not applicable to this article.

## Abbreviations

The following abbreviations are used in this manuscript:

HA	hyaluronan/hyaluronic acid
HYADD-4	hexadecylamide derivative of hyaluronan
BDDE	1,4-butanediol diglycidyl ether
G′	storage modulus
G″	loss modulus
CD44	cluster of differentiation 44
RHAMM	receptor for hyaluronan-mediated motility
TLR	toll-like receptor
NF-κB	nuclear factor kappa B
IL-1β	interleukin-1 beta
MMP	matrix metalloproteinase
ROS	reactive oxygen species
OA	osteoarthritis
BMAC	bone marrow aspirate concentrate
PRP	platelet-rich plasma
RCT	randomized controlled trial
MRI	magnetic resonance imaging
ADAMTS5	a disintegrin and metalloproteinase with thrombospondin motifs 5
DSC	differential scanning calorimetry
IKDC	International Knee Documentation Committee
KOOS	Knee injury and Osteoarthritis Outcome Score
NASHA	non-animal stabilized hyaluronic acid
NSAID	non-steroidal anti-inflammatory drug
PIIANP	procollagen type II N-terminal propeptide
TGA	thermogravimetric analysis
VAS	visual analogue scale
WOMAC	Western Ontario and McMaster Universities Osteoarthritis Index

## References

[1] Fraser J.R.E., Laurent T.C., Laurent U.B.G. (1997). Hyaluronan: Its Nature, Distribution, Functions and Turnover. J. Intern. Med..

[2] Hoffman A.S. (2002). Hydrogels for Biomedical Applications. Adv. Drug Deliv. Rev..

[3] Burdick J.A., Prestwich G.D. (2011). Hyaluronic Acid Hydrogels for Biomedical Applications. Adv. Mater..

[4] Altman R.D., Manjoo A., Fierlinger A., Niazi F., Nicholls M. (2015). The Mechanism of Action for Hyaluronic Acid Treatment in the Osteoarthritic Knee: A Systematic Review. BMC Musculoskelet. Disord..

[5] Flory P.J. (1941). Molecular Size Distribution in Three Dimensional Polymers. I. Gelation. J. Am. Chem. Soc..

[6] Stockmayer W.H. (1943). Theory of Molecular Size Distribution and Gel Formation in Branched-Chain Polymers. J. Chem. Phys..

[7] Winter H.H., Chambon F. (1986). Analysis of Linear Viscoelasticity of a Crosslinking Polymer at the Gel Point. J. Rheol..

[8] Winter H.H. (1987). Can the Gel Point of a Cross-Linking Polymer Be Detected by the G′–G″ Crossover?. Polym. Eng. Sci..

[9] Reiner M. (1964). The Deborah Number. Phys. Today.

[10] (2021). Fidia Farmaceutici S.p.A. Hymovis (High Molecular Weight Viscoelastic Hyaluronan HYADD4) Package Insert, 68573-13-C.

[11] Bustamante-Torres M., Romero-Fierro D., Arcentales-Vera B., Palomino K., Magaña H., Bucio E. (2021). Hydrogels Classification According to the Physical or Chemical Interactions and as Stimuli-Sensitive Materials. Gels.

[12] Finelli I., Chiessi E., Galesso D., Renier D., Paradossi G. (2011). A New Viscosupplement Based on Partially Hydrophobic Hyaluronic Acid: A Comparative Study. Biorheology.

[13] La Gatta A., Salzillo R., Catalano C., Pirozzi A.V.A., D’Agostino A., Bedini E., Cammarota M., De Rosa M., Schiraldi C. (2020). Hyaluronan-Based Hydrogels via Ether-Crosslinking: Is HA Molecular Weight an Effective Means to Tune Gel Performance?. Int. J. Biol. Macromol..

[14] Khunmanee S., Jeong Y., Park H. (2017). Crosslinking Method of Hyaluronic-Based Hydrogel for Biomedical Applications. J. Tissue Eng..

[15] Finelli I., Chiessi E., Galesso D., Renier D., Paradossi G. (2009). Gel-Like Structure of a Hexadecyl Derivative of Hyaluronic Acid for the Treatment of Osteoarthritis. Macromol. Biosci..

[16] de Gennes P.G. (1979). Scaling Concepts in Polymer Physics.

[17] Krause W.E., Bellomo E.G., Colby R.H. (2001). Rheology of Sodium Hyaluronate under Physiological Conditions. Biomacromolecules.

[18] Baek J., Fan Y., Jeong S.-H., Lee H.-Y., Jung H.-D., Kim H.-E., Kim S., Jang T.-S. (2018). Facile Strategy Involving Low-Temperature Chemical Cross-Linking to Enhance the Physical and Biological Properties of Hyaluronic Acid Hydrogel. Carbohydr. Polym..

[19] Zerbinati N., Sommatis S., Maccario C., Capillo M.C., Grimaldi G., Alonci G., Rauso R., Guida S., Mocchi R. (2021). Comparative Physicochemical Analysis among 1,4-Butanediol Diglycidyl Ether Cross-Linked Hyaluronic Acid Dermal Fillers. Gels.

[20] Wojtkiewicz M., Stachura A., Roszkowski B., Winiarska N., Kazimierska K., Stachura K. (2024). Are We Overlooking Harms of BDDE-Cross-Linked Dermal Fillers? A Scoping Review. Aesthetic Plast. Surg..

[21] Daminato E., Bianchini G., Causin V. (2022). New Directions in Aesthetic Medicine: A Novel and Hybrid Filler Based on Hyaluronic Acid and Lactose Modified Chitosan. Gels.

[22] Sadeghi H., Espino D.M., Shepherd D.E.T. (2015). Variation in Viscoelastic Properties of Bovine Articular Cartilage below, up to and above Healthy Gait-Relevant Loading Frequencies. Proc. Inst. Mech. Eng. Part H..

[23] Jahn S., Seror J., Klein J. (2016). Lubrication of Articular Cartilage. Annu. Rev. Biomed. Eng..

[24] Antonacci J.M., Schmidt T.A., Serventi L.A., Cai M.Z., Shu Y.L., Schumacher B.L., McIlwraith C.W., Sah R.L. (2012). Effects of Equine Joint Injury on Boundary Lubrication of Articular Cartilage by Synovial Fluid: Role of Hyaluronan. Arthritis Rheum..

[25] Yamazaki K., Fukuda K., Matsukawa M., Hara F., Matsushita T., Yamamoto N., Yoshida K., Munakata H., Hamanishi C. (2003). Cyclic Tensile Stretch Loaded on Bovine Chondrocytes Causes Depolymerization of Hyaluronan: Involvement of Reactive Oxygen Species. Arthritis Rheum..

[26] Pavan M., Galesso D., Secchieri C., Guarise C. (2016). Hyaluronic Acid Alkyl Derivative: A Novel Inhibitor of Metalloproteases and Hyaluronidases. Int. J. Biol. Macromol..

[27] Brun P., Zavan B., Vindigni V., Schiavinato A., Pozzuoli A., Iacobellis C., Abatangelo G. (2012). In Vitro Response of Osteoarthritic Chondrocytes and Fibroblast-like Synoviocytes to a 500–730 kDa Hyaluronan Amide Derivative. J. Biomed. Mater. Res. Part B Appl. Biomater..

[28] Al-Sibani M., Al-Harrasi A., Neubert R.H.H. (2016). Study of the Effect of Mixing Approach on Cross-Linking Efficiency of Hyaluronic Acid-Based Hydrogel Cross-Linked with 1,4-Butanediol Diglycidyl Ether. Eur. J. Pharm. Sci..

[29] Maiz-Fernández S., Pérez-Álvarez L., Ruiz-Rubio L., Pérez González R., Sáez-Martínez V., Ruiz Pérez J., Vilas-Vilela J.L. (2019). Synthesis and Characterization of Covalently Crosslinked pH-Responsive Hyaluronic Acid Nanogels: Effect of Synthesis Parameters. Polymers.

[30] Cassuto D., Bellia G., Schiraldi C. (2021). An Overview of Soft Tissue Fillers for Cosmetic Dermatology: From Filling to Regenerative Medicine. Clin. Cosmet. Investig. Dermatol..

[31] Chuysinuan P., Thanyacharoen T., Thongchai K., Techasakul S., Ummartyotin S. (2020). Preparation of Chitosan/Hydrolyzed Collagen/Hyaluronic Acid Based Hydrogel Composite with Caffeic Acid Addition. Int. J. Biol. Macromol..

[32] Wende F.J., Xue Y., Nestor G., Öhrlund Å., Sandström C. (2020). Relaxation and Diffusion of Water Protons in BDDE Cross-Linked Hyaluronic Acid Hydrogels Investigated by NMR Spectroscopy—Comparison with Physicochemical Properties. Carbohydr. Polym..

[33] Altman R.D., Dasa V., Takeuchi J. (2018). Review of the Mechanism of Action for Supartz FX in Knee Osteoarthritis. Cartilage.

[34] Avenoso A., D’Ascola A., Scuruchi M., Mandraffino G., Calatroni A., Saitta A., Campo S., Campo G.M. (2018). Hyaluronan in the Experimental Injury of the Cartilage: Biochemical Action and Protective Effects. Inflamm. Res..

[35] Smith M.M., Russell A.K., Schiavinato A., Little C.B. (2013). A Hexadecylamide Derivative of Hyaluronan (HYMOVIS) Has Superior Beneficial Effects on Human Osteoarthritic Chondrocytes and Synoviocytes than Unmodified Hyaluronan. J. Inflamm..

[36] Smith M.M., Cake M.A., Ghosh P., Schiavinato A., Read R.A., Little C.B. (2008). Significant Synovial Pathology in a Meniscectomy Model of Osteoarthritis: Modification by Intra-Articular Hyaluronan Therapy. Rheumatology.

[37] Cake M.A., Read R.A., Edwards S., Smith M.M., Burkhardt D., Little C.B., Ghosh P. (2008). Changes in Gait after Bilateral Meniscectomy in Sheep: Effect of Two Hyaluronan Preparations. J. Orthop. Sci..

[38] Balazs E.A., Denlinger J.L. (1993). Viscosupplementation: A New Concept in the Treatment of Osteoarthritis. J. Rheumatol. Suppl..

[39] Sun S.-F., Hsu C.-W., Lin H.-S., Liou I.-H., Chen Y.-H., Hung C.-L. (2017). Comparison of Single Intra-Articular Injection of Novel Hyaluronan (HYA-JOINT Plus) with Synvisc-One for Knee Osteoarthritis: A Randomized, Controlled, Double-Blind Trial of Efficacy and Safety. J. Bone Jt. Surg. Am..

[40] Ko P.-Y., Li C.-Y., Li C.-L., Kuo L.-C., Su W.-R., Jou I.-M., Wu P.-T. (2022). Single Injection of Cross-Linked Hyaluronate in Knee Osteoarthritis: A 52-Week Double-Blind Randomized Controlled Trial. Pharmaceutics.

[41] Bashaireh K., Naser Z., Hawadya K.A., Sorour S., Al-Khateeb R.N. (2015). Efficacy and Safety of Cross-Linked Hyaluronic Acid Single Injection on Osteoarthritis of the Knee: A Post-Marketing Phase IV Study. Drug Des. Devel. Ther..

[42] Priano F. (2017). Early Efficacy of Intra-Articular HYADD 4 (Hymovis) Injections for Symptomatic Knee Osteoarthritis. Joints.

[43] Bisicchia S., Bernardi G., Tudisco C. (2016). HYADD 4 versus Methylprednisolone Acetate in Symptomatic Knee Osteoarthritis: A Single-Centre Single Blind Prospective Randomised Controlled Clinical Study with 1-Year Follow-Up. Clin. Exp. Rheumatol..

[44] Benazzo F., Perticarini L., Padolino A., Castelli A., Gifuni P., Lovato M., Manzini C., Giordan N. (2016). A Multi-Centre, Open Label, Long-Term Follow-Up Study to Evaluate the Benefits of a New Viscoelastic Hydrogel (Hymovis) in the Treatment of Knee Osteoarthritis. Eur. Rev. Med. Pharmacol. Sci..

[45] Tamburrino P., Castellacci E. (2016). Intra-Articular Injections of HYADD4-G in Male Professional Soccer Players with Traumatic or Degenerative Knee Chondropathy. A Pilot, Prospective Study. J. Sports Med. Phys. Fit..

[46] Zorzi C., Rigotti S., Screpis D., Giordan N., Piovan G. (2016). A New Hydrogel for the Conservative Treatment of Meniscal Lesions: A Randomized Controlled Study. Joints.

[47] Henrotin Y., Bannuru R., Malaise M., Ea H.-K., Confavreux C., Bentin J., Urbin-Choffray D., Conrozier T., Brasseur J.-P., Thomas P. (2019). Hyaluronan Derivative HYMOVIS Increases Cartilage Volume and Type II Collagen Turnover in Osteoarthritic Knee: Data from MOKHA Study. BMC Musculoskelet. Disord..

[48] Mainil-Varlet P., Schiavinato A., Ganster M.M. (2013). Efficacy Evaluation of a New Hyaluronan Derivative HYADD 4-G to Maintain Cartilage Integrity in a Rabbit Model of Osteoarthritis. Cartilage.

[49] Duan X., Sandell L.J., Chinzei N., Holguin N., Silva M.J., Schiavinato A., Rai M.F. (2017). Therapeutic Efficacy of Intra-Articular Hyaluronan Derivative and Platelet-Rich Plasma in Mice Following Axial Tibial Loading. PLoS ONE.

[50] Gobbi A., Whyte G.P. (2016). One-Stage Cartilage Repair Using a Hyaluronic Acid-Based Scaffold with Activated Bone Marrow-Derived Mesenchymal Stem Cells Compared with Microfracture: Five-Year Follow-Up. Am. J. Sports Med..

[51] Gobbi A., Whyte G.P. (2019). Long-Term Clinical Outcomes of One-Stage Cartilage Repair in the Knee with Hyaluronic Acid-Based Scaffold Embedded with Mesenchymal Stem Cells Sourced from Bone Marrow Aspirate Concentrate. Am. J. Sports Med..

[52] Whyte G.P., Bizzoco L., Gobbi A. (2024). One-Step Cartilage Repair of Full-Thickness Knee Chondral Lesions Using a Hyaluronic Acid-Based Scaffold Embedded with Bone Marrow Aspirate Concentrate: Long-Term Outcomes after Mean Follow-Up Duration of 14 Years. Am. J. Sports Med..

[53] Kim I.L., Mauck R.L., Burdick J.A. (2011). Hydrogel Design for Cartilage Tissue Engineering: A Case Study with Hyaluronic Acid. Biomaterials.

[54] Heirani-Tabasi A., Hosseinzadeh S., Rabbani S., Ahmadi Tafti S.H., Jamshidi K., Soufizomorrod M., Soleimani M. (2021). Cartilage Tissue Engineering by Co-Transplantation of Chondrocyte Extracellular Vesicles and Mesenchymal Stem Cells, Entrapped in Chitosan-Hyaluronic Acid Hydrogel. Biomed. Mater..

[55] Dong Q., Zhong X., Zhang Y., Bao B., Liu L., Bao H., Bao C., Cheng X., Zhu L., Lin Q. (2020). Hyaluronic Acid-Based Antibacterial Hydrogels Constructed by a Hybrid Crosslinking Strategy for Pacemaker Pocket Infection Prevention. Carbohydr. Polym..

[56] Ishihara K. (2025). Advances in Bioinspired Polymer Hydrogel Systems with Biomedical Functionalities. Sci. Technol. Adv. Mater..

